# Folate system correlations in DNA microarray data

**DOI:** 10.1186/1471-2407-5-95

**Published:** 2005-08-04

**Authors:** Tomas Radivoyevitch

**Affiliations:** 1 Department of Epidemiology and Biostatistics Case Western Reserve University, Cleveland, Ohio 44106 USA

## Abstract

**Background:**

Gene expression data is abundantly available from the Gene Expression Omnibus (GEO) and various websites. Pathway specific analyses of gene-gene correlations across these datasets remain relatively unexplored, though they could be informative.

**Methods:**

Folate gene expression data is explored here in two ways: (1) directly, using gene-gene scatter plots and gene expression time course plots; and (2) indirectly, using de novo purine synthesis (DNPS) and de novo thymidylate synthesis (DNTS) flux predictions of a folate model perturbed by relative gene expression modulations of its V_max _parameters.

**Results:**

Positive correlations within and between the DNPS and DNTS folate cycles are observed in the folate gene expression data. For steady state measurements across childhood leukemia patients, positive correlations between DNPS and DNTS are consistent with higher proliferative fractions requiring higher levels of both fluxes. For cells exposed to ionizing radiation, transient increases in both pathways are consistent with DNA damage driven dNTP demand, and a steadily decreasing backdrop is consistent with radiation induced cell cycle arrest. By and large, folate model based flux predictions paralleled these findings, the main differences being a gain of correlation information for the TEL-AML1 leukemia data, and the loss of one interesting inference, namely, that RNA repair driven DNPS precedes DNA repair driven DNTS after a 10 gray dose of ionizing radiation.

**Conclusion:**

Pathway focused correlation analyses of DNA microarray data can be informative, with or without a mathematical model. Conceptual models are essential. Mathematical model based analyses should supplement, but should not replace, direct data analyses.

## Background

**Figure 1:**
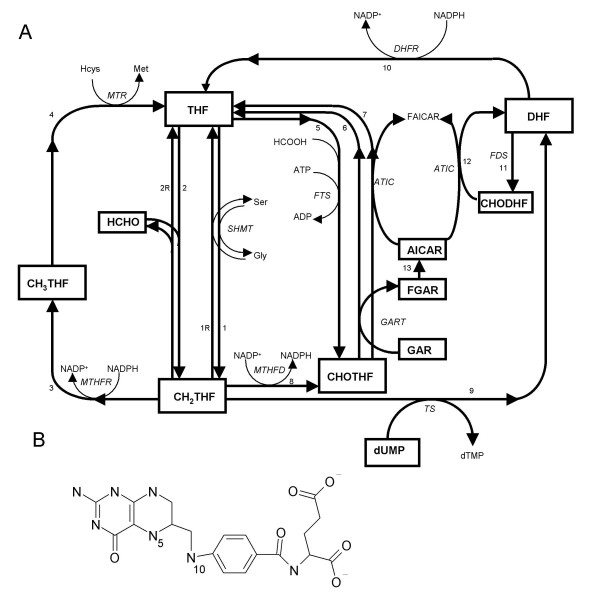
The folate cycle model of Morrison and Allegra [1] (A) and the molecular structure of tetrahydrofolate (B).

The folate system (Figure 1A) is central to *de novo *purine synthesis (DNPS) and *de novo *thymidylate synthesis (DNTS) and is a key target of several anti-cancer agents. For example, methotrexate (MTX), in its polyglutamated forms, inhibits dihydrofolate [DHF] reductase (DHFR), thymidylate synthetase (TS), glycinamide ribonucleotide formyltransferase (GART), and other folate system enzymes (see the MTX containing reaction equations [1] in Methods); the novel multi-targeted anti-folate ALIMTA has similar targets [2] though with a very different spectrum of inhibition constants *K*_i _such that GART inhibition is dominant [3]; the anti-folate raltitrexed (Tomudex) mostly inhibits TS [4]; and 5-fluorouracil also inhibits TS (as FdUMP), though it also kills cells via incorporation into DNA and RNA [5].

Folates convert serine side chains into tetrahydrofolate (THF; Figure 1B) held reactive single-carbons with the following uses: 5-methyl-THF (CH_3_THF) is used to convert homocysteine into methionine; 5,10 methylene-THF (CH_2_THF) is used by TS of DNTS to convert dUMP (deoxyuridylate) into dTMP (thymidylate) for the sole purpose of DNA synthesis, be it scheduled (i.e. DNA replication driven) or unscheduled (i.e. DNA damage driven); and 10-formyl-THF (CHOTHF) is used to set up the purine ring closure reactions of DNPS for many purposes, including the synthesis of RNA, DNA, ATP, GTP and many other molecules, all of which are subject to purine base oxidation and thus replacement after irradiation. These three single-carbon consuming folate functions interact via competition for CH_2_THF. Using publicly available DNA microarray data, this study explores folate cycle interactions at the higher level of mRNA. To assess the added value to analyses of a published mathematical model of the folate system [1], inferences obtained directly from the DNA microarray data are compared here to those obtained indirectly using folate model predictions of DNPS and DNTS based on the same DNA microarray data.

## Methods

### Mathematical model of Morrison and Allegra

**Figure 2:**
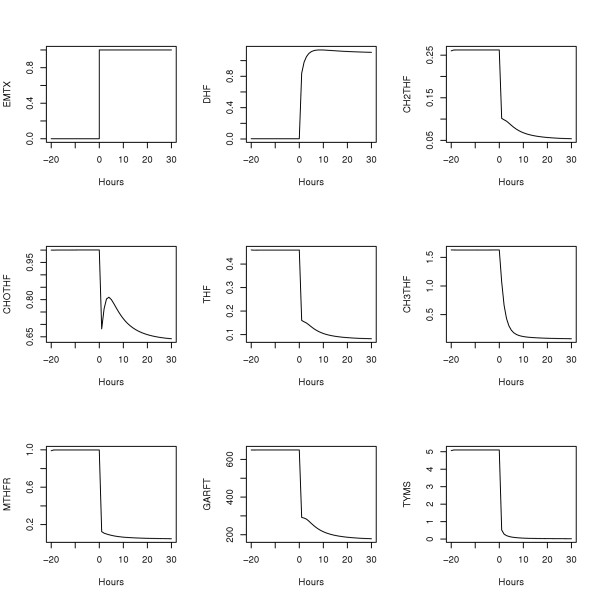
Morrison and Allegra's model [1] responding to 1 μM MTX applied continuously after t = 0. Concentrations are in μM (top 6 plots) and fluxes are in μM/hour (bottom 3 plots).

The folate cycle model of Morrison and Allegra [1] has the following mathematical form:



These equations restate the system configuration information of Figure 1A, i.e. they state that the rate at which a metabolite concentration increases equals the sum of the synthesis reaction fluxes (arrows into a node) minus the sum of the degradation reaction fluxes (arrows leaving a node). The *r*_i _in these equations are:



where DHF^T^-DHF (total DHF minus free DHF) is the concentration of DHF bound to DHFR, x_i _is the concentration of *i*-glutamated MTX, and all folates are assumed to be penta-glutamated. Not shown are 10 additional differential equations for up to penta-glutamation of MTX either free or bound to DHFR. These 10 equations are irrelevant when MTX = 0 as in the microarray data analyses below; they were used here only to validate the current implementation of the model against its previously published responses, see Figure 2[1].

### Model limitations

Since the model has only one compartment, the cytosol, it cannot handle changes in the mitochondrial enzymes MTHFD2 and SHMT2, nor can it handle changes in the extra-cellular folate hydrolase gene FOLH1 (the gene that codes for prostate-specific membrane antigen, PSMA), so these genes were ignored in the analyses. Further, the folate genes GGH (polyglutamate hydrolase), FPGS (polyglutamate synthase), and RFC (reduced folate carrier), were not considered in the microarray analyses because these reactions are included in the model only for MTX and not folate, and because MTX = 0 for the microarray data.

### Model modifications

Flux boundary conditions for dUMP and GAR synthesis in the original model [1] were replaced by downstream concentration boundary conditions set to their initial values. This was done because *steady state *flux differences across patients would otherwise be nullified; e.g. if the flux into GAR were fixed, the steady state flux through GART would also be fixed, and artificially then, there would be no variation in predictions of this flux across patients.

### Model-data linkage

For steady state flux predictions of leukemia patient diagnostic samples, MAS5 microarray measurements of Ross et al [6] and Yeoh et al [7] were normalized by dividing by the mean of the leukemia subtype medians. Step functions from 1 (for *t *< 0) to the resulting metrics (for *t *≥ 0) were then used as modulators of the baseline folate model V_max _values, i.e. microarray data normalization values were equated to the steady state of the model. Individualized patient steady states were then computed as simulation endpoints 40 hours after the V_max _perturbations; all time courses were inspected visually to assure settled steady states at 40 hours. For radiation response data [8], initial measurements were equated to the steady state of the model, so the data was normalized by its values at *t *= 0. Linear interpolations of the normalized data were then used as time-varying V_max _modulators. For both the steady state leukemia data and the time course radiation response data, proportionality between mRNA levels and protein levels was assumed. This assumption was motivated by simplicity and a lack of better alternatives. As proteomic-transcriptomic combined dynamic response data (e.g. [9]) accrues, it will likely be replaced by a set of gene specific lead-lag filter [10] assumptions. In the meantime, it can be viewed and used as a first order approximation, or as a temporary crutch.

### Computational details

**Figure 3:**
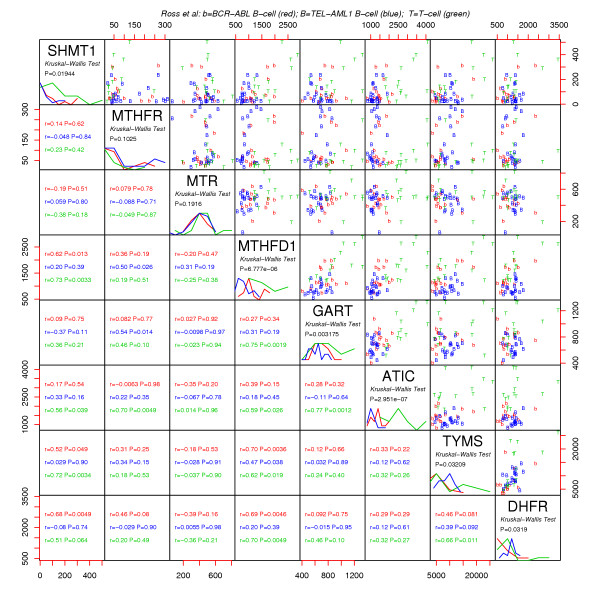
MAS5 U133a folate gene expression data of Ross et al [7]. Symbols are TEL-AML1 (B), BCR-ABL (b) and T-cell (T). In mirrored positions relative to the main diagonal, corresponding Pearson correlation coefficients *r *are given with their P values.

**Figure 4:**
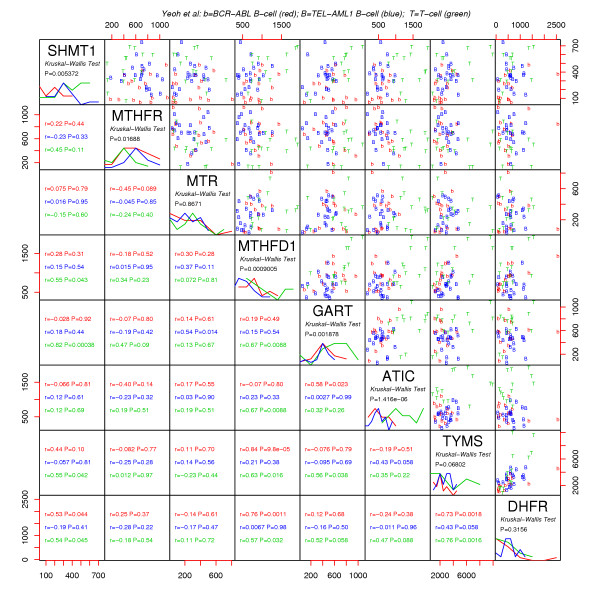
MAS5 U95av2 folate gene expression data of Yeoh et al [8]. Only the Yeoh et al [8] patients who are also in the Ross et al dataset [7] are considered. MAS5 summary measures were computed from cel files using Bioconductor's AFFY package. Symbols are as in Figure 3.

**Figure 5:**
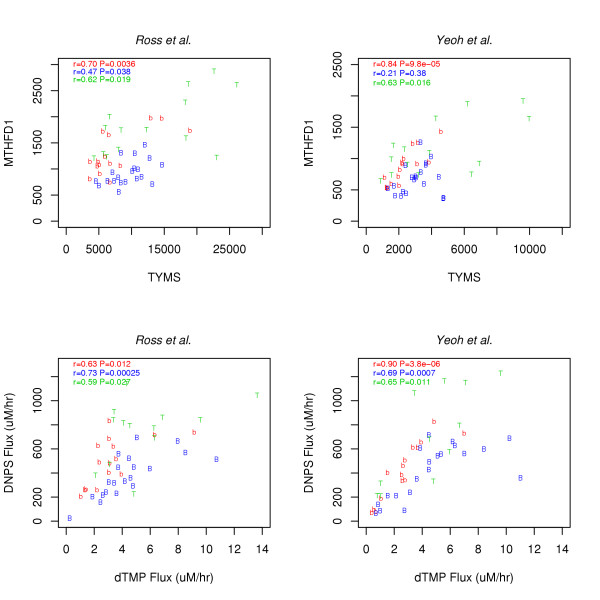
Comparisons of measured MTHFD1 vs. TYMS versus predicted DNPS vs. DNTS.

**Figure 6:**
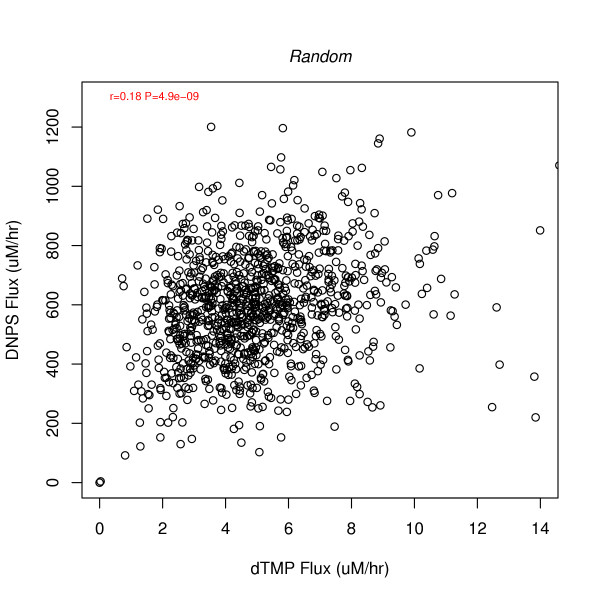
DNPS vs. DNTS predicted with random numbers replacing Figures 3 and 4.

**Figure 7:**
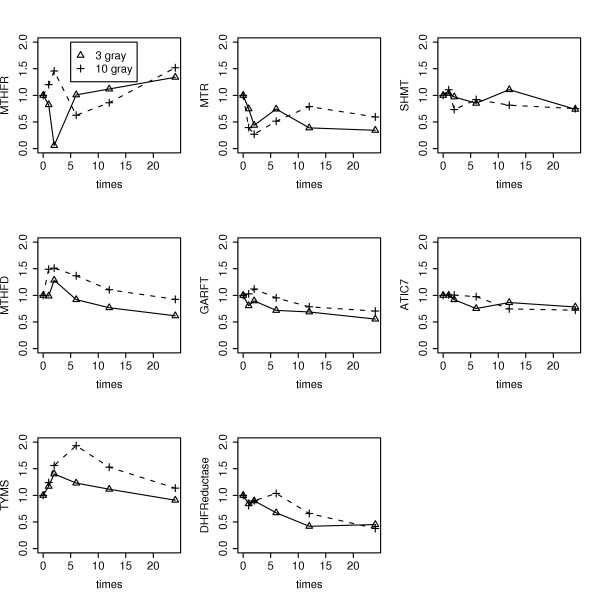
Lymphocyte radiation response data of Jen and Cheung [8]. SHMT1 and MTHFR were not applied to the folate model, see text. Time is in hours.

**Figure 8:**
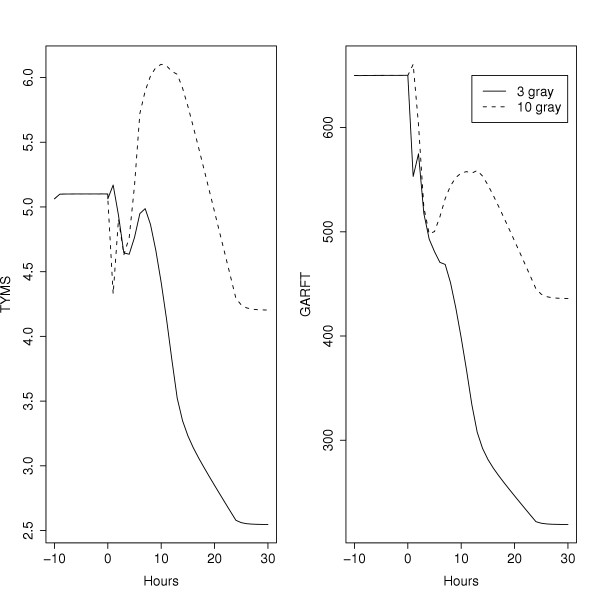
The folate model's response to the radiation time course data. Fluxes are in μM/hr.

The computational environment R [11] was used with R packages of Bioconductor [12] to implement this study. Specifically, the package Biobase was used to manipulate microarray data as expression set (class eset) objects and SBMLR [13,14] was used to simulate a systems biology markup language (SBML) [15-17] representation of the folate metabolism model [1]. R scripts reproducing figures 2, 3, 4, 5, 6, 7, 8 are included with SBMLR as an illustrative example of package use. For convenience, array data used in this study have been repackaged as eset objects in R data packages available from the author's website [14]. Throughout, genes with multiple probe sets were represented by the set with the highest average value.

## Results

### Folate system correlations across childhood leukemias

Childhood acute lymphoblastic leukemia (ALL) microarray data of Ross et al [6] and Yeoh et al [7] is shown in Figures 3 and 4. Several points can be made regarding this steady state diagnostic bone marrow data. Firstly, since TYMS and DHFR (similarly MTHFD1, GART and ATIC) operate in series, it makes sense that the system would attempt to match these throughput capabilities as closely as possible to avoid the costs of maintaining unneeded excess "equipment." Thus, positive correlations *within *the DNPS and DNTS branches are expected. Secondly, growing cells require commensurate increases in both DNTSand DNPS, so positive correlations *between *these cycles are also expected. Finally, DNPS genes are higher in T cell leukemic cells than in B-cell leukemic cells, consistent with measured DNPS fluxes being three fold higher in T cells than in B cells [18].

To assess the added value of the folate model, since MTHFD1 and TYMS are the gatekeepers of DNPS and DNTS, respectively, correlation plots for these genes were compared to corresponding flux predictions in Figure 5. The plots show that, for the most part, model predicted fluxes are more correlated than measured MTHFD1 vs. TYMS mRNA. To estimate the amount of correlation attributable to steady state flux constraints alone, 1000 uncorrelated normally distributed (μ = 1; σ = 0.30) random numbers were applied to the model as V_max _modulators. The amount of correlation (*r *= 0.18, Figure 6) is more than that induced by the model (beyond gatekeeper correlations) for BCR-ABL and T cell leukemias (Figure 5), but less than induced for TEL-AML1 leukemias. Thus, for TEL-AML leukemias additional information must have been contributed by the other folate genes inputted into the model, suggesting more folate system coordination in this more curable leukemia subtype.

### Folate system analysis of radiation time course data

Folate system correlations in radiation response time course data [8] were also investigated. The data (Fig. 7) shows that TS, DHFR, GARFT and MTHFD each have a dose-dependent transient increase after irradiation, consistent with radiation induced DNA damage causing a transient rise and fall in P53 activity [19] with subsequent induction of ribonucleotide reductase subunit P53R2 [20] causing a transient increase in *de novo *deoxynucleotide synthesis for DNA repair; a 17-fold increase in R2 protein 24 h after irradiation [21] is radioprotective [22], further supporting this conjecture. The data also shows a steady decline in many of the gene expression time courses, possibly due to radiation induced cell cycle arrest. At 10 gray, inspection of TS, DHFR, GARFT and MTHFD further suggests that RNA repair driven DNPS (peak at 2 hours) precedes DNA repair driven DNTS (peak at 6 hours).

In Figure 7, gene expression time courses are more likely to be signals if they differ between 3 and 10 gray only in terms of minor time shifts and dose ordered amplitude changes. Based on this, MTHFR and SHMT1 were dismissed as noise. The remaining six gene expression time courses were normalized by their values at *t *= 0 and applied to the folate model as linearly interpolated time-varying modulators of corresponding *V*_max _parameters. The resulting model-based predicted time courses are shown in Figure 8. These plots affirm the "dose-dependent spike resting on a decreasing backdrop" response of DNPS and DNTS that was qualitatively inferred from Figure 7. In contrast to the data itself, however, at 10 gray, RNA repair driven DNPS (peak at 12 hours) and DNA repair driven DNTS (peak at 10 hours) are reverse ordered and delayed. Since NTP production tends to be in high gear full time, compared to dNTP production, it likely has a greater ability to respond rapidly to oxidative damage. Further, a dNTP synthesis flux peak at 6 hours compared to 10 hours is more consistent with a P53 spike at 5 hours [19]. Thus, until measurements of DNPS and DNTS suggest otherwise, the data-based inference that RNA repair precedes DNA repair is tentatively more credible than its model-based counterpart.

## Discussion

Pathway focused analyses are essential for gene-gene correlation studies because the number of possible correlations would otherwise be too large to investigate. For example, if a chip carries 10,000 genes, the number of 2D plots requiring correlation tests is then 10,000 choose 2, or ~50 million, i.e. a multiple testing problem not encountered in pathway focused studies.

Although TS and DHFR are predominantly controlled at the level of protein translation [23], Figures 3, 4 and 7 indicate that some control effort is also exerted at the mRNA level. Thus, protein level control may dominate a particular regulatory system, but mRNA signals may still be informative of what the overall system is trying to accomplish. That DNPS and DNTS are correlated in a consistent manner across disparate steady state-and transient datasets lends credence to the view that DNA microarray data is a valid source of biochemical system coordination and control information. Characterization of cancer differences in coordination and control could be relevant to future treatment designs and should thus be further explored.

## Conclusion

The main conclusion of this paper is that interesting inferences can be gleaned from genome-wide microarray data (with or without mathematical models) if gene-gene correlations are analyzed in a pathway specific manner. The added value of analyzing microarray data using Morrison and Allegra's folate model, relative to simply *eye-balling *the gene expression data, was minimal. For example, in Figure 5, save the model's ability to identify TEL-AML1 leukemias as being additionally coordinated, gate-keeper focused gene expression scatter plots are almost as revealing as model-predicted DNPS vs. DNTS scatter plots. Similarly, for the radiation time course data in Figures 7 and 8, the spike increase in DNPS and DNTS and the baseline downward trend at larger times are apparent using either approach.

This study used conceptual models of folates and the biological effects of ionizing radiation to guide, focus, validate and discriminate microarray data inferences. Further, knowledge of system scope was used to reduce the analysis to a manageable dimension correlated subspace. This suggests that pathway focused analyses are more likely to be successful if they are applied to biochemical systems and experimental perturbations that are well understood.

To go beyond qualitative statements and to actually plot predictions (Figures 5 and 8), quantitative models are needed. Further, as biochemical system knowledge expands in scope and complexity, *gedanken *experiments underlying *eye-ball *data analyses will become increasingly difficult to carry out. Thus, model-based approaches must continue to be developed. At the same time, since the inference that RNA repair precedes DNA repair was lost in the model-based approach, such approaches should supplement, but should not replace, direct data analyses.

## Abbreviations

DHF = dihydrofolate; THF = tetrahydrofolate; DNPS = de novo purine synthesis; DNTS = de novo thymidylate synthesis; DHFR = DHF reductase; TS = thymidylate synthetase (protein); TYMS = thymidylate synthetase (gene); MTHFR = methylene-THF reductase; MTR = methyl-THF transferase; FTS = formyl-THF synthetase; FDS = formyl-DHF synthetase; SHMT = serine hydroxy methyl transferase; GAR = glycinamide ribonucleotide; FGAR = formyl-GAR; GART = GAR formyltransferase; AICAR = aminoimidazole-4-carboxamide ribonucleotide; FAICAR = formyl-AICAR; dUMP = deoxyuridylate; dTMP = thymidylate; MTX = methotrexate; SBML = Systems Biology Markup Language.

## Competing interests

The author declares that he has no competing interests.

## Authors' contributions

The author is the sole contributor.

## Pre-publication history

The pre-publication history for this paper can be accessed here:
